# Is crossed laterality associated with academic achievement and intelligence? A systematic review and meta-analysis

**DOI:** 10.1371/journal.pone.0183618

**Published:** 2017-08-28

**Authors:** Marta Ferrero, Gillian West, Miguel A. Vadillo

**Affiliations:** 1 Department of Experimental Psychology, University College London, London,United Kingdom; 2 Facultad de Ingeniería, Universidad de Deusto, Bilbao, Spain; 3 Department of Language and Cognition, University College London, London, United Kingdom; 4 Primary Care and Public Health Sciences, King’s College London, London, United Kingdom; 5 Departamento de Psicología Básica, Universidad Autónoma de Madrid, Madrid, Spain; Universitat Wien, AUSTRIA

## Abstract

Over the last century, sporadic research has suggested that people whose hand, eye, foot, or ear dominances are not consistently right- or left-sided are at special risk of suffering academic difficulties. This phenomenon is known as crossed laterality. Although the bulk of this research dates from 1960’s and 1970’s, crossed laterality is becoming increasingly popular in the area of school education, driving the creation of several interventions aimed at restoring or consolidating lateral dominance. However, the available evidence is fragmentary. To determine the impact of crossed laterality on academic achievement and intelligence, we conducted a systematic review and meta-analysis of articles published since 1900. The inclusion criteria for the review required that studies used one or more lateral preference tasks for at least two specific parts of the body; they included a valid measure of crossed laterality; they measured the impact of crossed laterality on academic achievement or intelligence; and they included participants between 3 and 17 years old. The final sample included 26 articles that covered a total population of 3578 children aged 5 to 12. Taken collectively, the results of these studies do not support the claim that there is a reliable association between crossed laterality and either academic achievement or intelligence. Along with this, we detected important shortcomings in the literature, such as considerable heterogeneity among the variables used to measure laterality and among the tasks utilized to measure the outcomes. The educational implications of these results are discussed.

## Introduction

The term “crossed laterality” is employed to refer to people whose hand, eye, foot, or ear dominance are not uniformly right- or left-sided. The idea that crossed laterality is linked to academic performance is becoming increasingly popular in the area of school education. A simple search in Google of the terms "Crossed + laterality + right + hand + left + eye + exercises" returns around 33.000 entries, most of them containing links to articles, fora and specialised centres focused on this issue. The majority of these sites establish a direct association between crossed laterality and learning difficulties and offer information about different programmes aimed at remediating these disabilities by restoring unilateral dominance in children. For instance, The Institute for Neuro-Physiological Psychology (INPP), founded in the UK and now established in more than 20 countries, offers remedial training for children who show crossed laterality, with the aim of improving their educational outcome. It also offers licenses to use the INPP method as an independent practitioner. Similarly, The Brain Balance Achievement Centres, with around 100 subsidiaries in United States, offer a specific training called The Brain Balance Program, aimed at restoring natural dominance in children with a mixed-dominance profile. Finally, The Superior Institute of Psychological Studies offers assessments and interventions for crossed laterality in more than 15 centres in Spain. These programs may cost up to €350 [1].

Since Orton [2] suggested that an ill-established cerebral dominance was the cause of several disabilities, a number of interventions, such as the ones mentioned above, have been created to ameliorate learning disabilities by performing specific physical exercises that are assumed to restore or consolidate laterality. For instance, a popular method known as patterning [3] is aimed at imposing cerebral dominance through a series of physical exercises which consist of manipulating the child’s head and/or extremities in patterns intended to imitate prenatal and postnatal movements. The method lacks any supporting evidence and has been the object of severe criticism [4,5]. However, it remains popular in several countries [6]. Another intervention based to some extent on Orton’s theory is Brain Gym^®^, an educational program originally created by Paul E. Dennison and Gail Dennison in 1980. Brain Gym^®^ prescribes a number of simple movements intended to improve the integration of specific brain functions with body movements. According to its authors, lateral dominance is essential for reading, writing, listening, speaking, and the ability to move and think at the same time [7]. In spite of its popularity in schools all over the world, this intervention has been widely proven as ineffective [8].

Several studies have reported that crossed lateral people are at special risk of suffering learning disabilities, while others have reported the opposite result [9–18]. According to these mixed results, crossed laterality might or might not be a risk factor for learning disabilities and poor academic performance. In the same vein, studies addressing the relationship between handedness (regardless of lateral dominance in other parts of the body) and intelligence have yielded mixed and inconsistent results, although recent meta-analyses point to a slight cognitive advantage of right-handers [19] and a higher prevalence of atypical-handedness among intellectually disabled individuals [20]. In any case, to the best of our knowledge, the effectiveness of existing interventions aimed at addressing crossed laterality has never been put to the test in rigorous studies. The use of interventions with dubious or null evidence in the classroom or other educational contexts can be harmful in different ways. For instance, educational professionals and families must often devote much energy and emotion during the implementation of the interventions [21]. Many of these practices involve all the actors spending a great deal of time and enormous sums of money in individual counselling sessions for children, formative sessions for teachers and families or acquisition of reference materials by schools. This waste of time and money involves an opportunity cost, because these resources are taken away from effective treatments [22]. Even worse, the replacement of evidence-based practices by non-evidence based practices can harm children [23,24].

The fact that interventions aimed at addressing crossed laterality have not been properly tested does not necessarily mean that some of their basic tenets regarding the relationship between crossed laterality, intelligence and academic performance have not been explored. In fact, as previously mentioned, since the 1960’s a number of studies have tried to measure the impact of lateral dominance on these variables with mixed results [13,25]. The goal of the present study is to conduct a systematic review of all the available evidence to assess the impact of crossed laterality on academic achievement and intelligence among students, from kindergarten to high school. If a link between them exists, it would be imperative to design studies that tackle the effectiveness of the interventions utilized in school settings. By contrast, if no link is found, it would be reasonable to divert the available resources into testing the effectiveness of scientifically grounded interventions.

## Materials and methods

### Search procedures

The present systematic review follows the recommendations of the PRISMA (see S1 Table and [26]). On August 15th 2016 the first author (MF) conducted an electronic search on the *Web of Science* entering the terms “[(preference OR dominance) AND (hand OR eye OR ear OR foot OR manual OR ocular OR lateral OR cerebral)] OR [(cross*) AND (lateral* OR dominance OR preference)] OR [(mix*) AND (preference OR dominance)] OR [(hemispheric) AND (indecision OR specialization)] OR asymmetry OR bilateral OR laterality OR lateralization OR eyeness OR handedness OR hand skill” into the Title field. The search was limited to (a) English-language articles (b) published between 1900 and 2015 (c) with categories restricted to “behavioural sciences”, "neuroscience", "psychology", "psychology applied", "psychology biological", "psychology developmental", "psychology experimental", "psychology multidisciplinary" and "psychology social", and (d) research areas restricted to “behavioural sciences”, “education/educational research”, “neurosciences/neurology”, and “psychology”. Unpublished dissertations, reviews and meta-analysis were excluded at this stage. After removing 503 duplicates this initial search yielded a sample of 10,540 studies.

**Fig 1:**
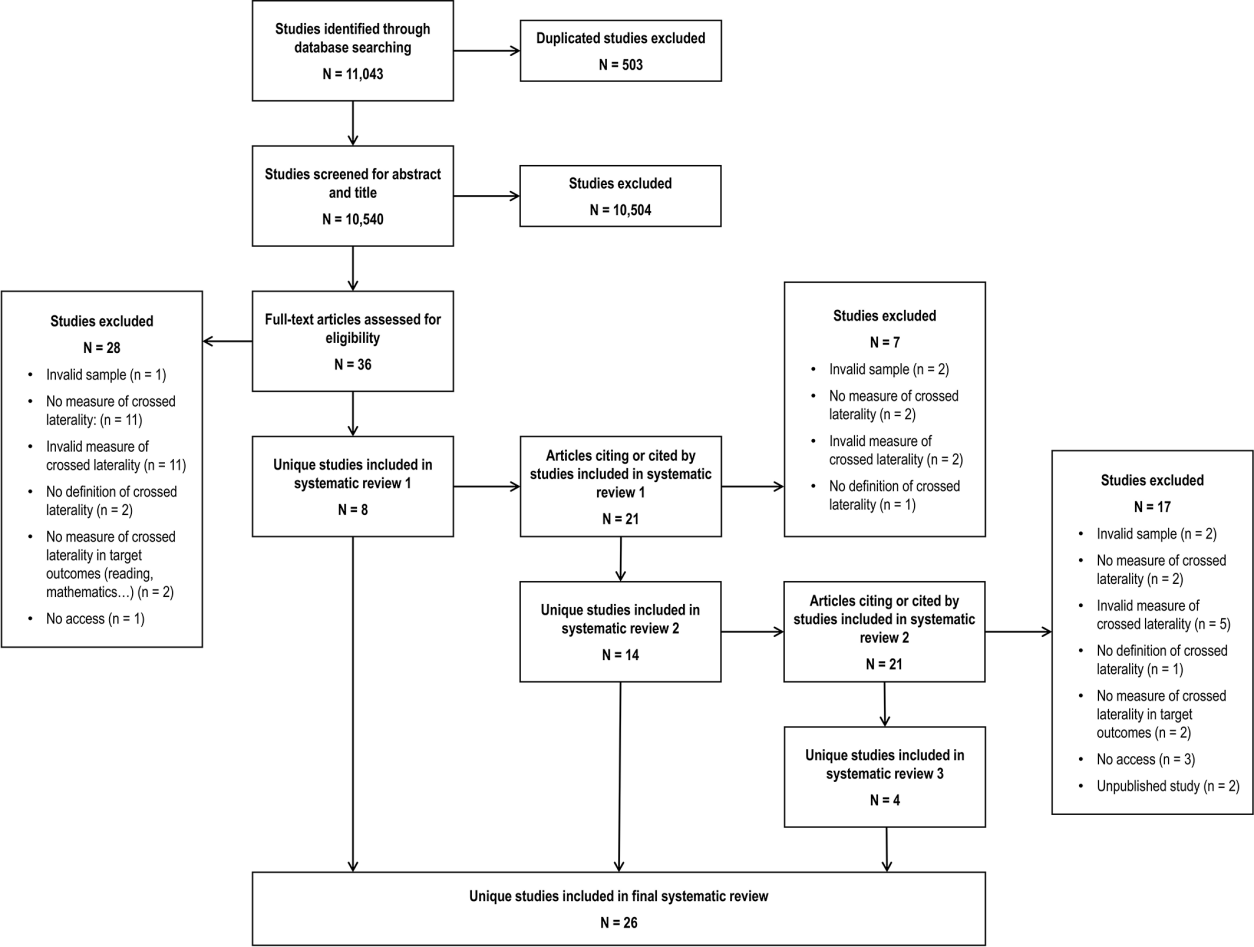
PRISMA flowchart.

Titles and abstracts of these studies were screened by MF using the inclusion criteria C1-C4 explained below. This resulted in 36 full-text articles assessed for eligibility. Authors MF and MAV independently read the full texts of these 36 articles to check whether they fulfilled the inclusion criteria. Among the initial set of 36 articles assessed for eligibility, eight articles complied with the inclusion criteria. Thereupon, descendancy searches of articles citing or cited by these eight papers were conducted to identify additional studies. This resulted in 21 full-text articles which were also read independently by MF and MAV. Among them, a total of 14 studies were selected by the inclusion criteria. A second round of descendancy searches was conducted for articles citing or cited by each of these 14 papers, which led to 21 extra articles assessed for eligibility. Among this set, 4 additional studies were selected. Therefore, the final sample of articles reviewed for inclusion comprised 26 articles (8 + 14 + 4). These articles [11,12,15,18,27–48] are shown in Table 1. A PRISMA flowchart summarizing the literature search process is depicted in Fig 1. Two of the studies selected were based on data that had already been collected and analysed in other articles, also included in our selection [28,29,40,41]. Across all the full-text articles read for inclusion, initial inter-rater agreement was 93.58%. Disagreements were resolved by discussion and consensus between the two researchers until there was 100% agreement.

**Table 1:** Articles that met inclusion and quality criteria.

Author; year	N	Gender	Age	Selection criteria	Setting	Laterality		Academic achievement				Intelligence
						Body part	Task	Reading	Spelling	Arithmetic	Language	
Annet & Turner, 1974 [27] [A]	224	120F 104M	60–132 months	school population	city nursery school and infant and junior schools	Hand, eye^1^, foot	non-standardised (^8^)	n.s.	**__**	**__**	The Peabody Picture Vocabulary Test	The maze test of the Wechsler Scale
Balow, 1963 [28] [A]	302	151F, 151M	84 months	school population	n.s.	hand, eye^1^	Test Harris (^8^)	The Gates Reading Readiness Tests Gates Primary Reading Tests PPR-Paragraph Reading	**__**	**__**	**__**	Lorge-Thorndike Intelligence Test
Balow & Barlow 1964 [29] [A]	250	n.s.	96 months	school population	n.s.	hand, eye^1^	Test Harris (^8^)	The Gates Reading Readiness Tests Gates Primary Reading Tests PPR-Paragraph Reading	**__**	**__**	**__**	Lorge-Thorndike Intelligence Test
Bishop et al. 1979 [30] [A]	147	n.s.	102 months	school population	rural general practice	hand, eye^2^	non-standardised (^8^)	Neale Reading Ability Test	**__**	**__**	**__**	Wechsler Scale for Children-R
Brod & Hamilton, 1971 [31] [AB]	54,51,52 (A,N,D)	n.s.	132 months	school population	parochial school	hand, eye^1^	non-standardised and the hole-in-the- card test(^8^)	Reading of passages	**__**	**__**	**__**	__
Bryden, 1970 [11] [B]	234	n.s.	84 months (GR2) 108 months (GR4) 132 months (GR6)	school population	n.s.	hand, ear^5^	non-standardised (^8^)	The Gates-MacGinitie Reading Test	**__**	**__**	**__**	The Otis Quick-Scoring Mental Ability Test
Clymer & Silva, 1985 [32] [A]	890	n.s.	84 months	all born in the same hospital	n.s.	hand, eye^1^, foot^4^	Test Harris (^8^)	__	**__**	**__**	The Dunedin Articulation Check The Illinois Test of Psycho-linguistic Abilities	Wechsler Scale for Children-R
Coleman & Deutsch, 1964 [33] [A]	121	7F, 28M (N) 56M (D) 26M, 4F (D)	108.5–144.3 months (D) 120.3–144 months (N)	reading disabilities	n.s.	hand, eye^1^, foot	Test Harris (^8^)	__	**__**	**__**	**__**	__
Conolly, 1983 [34] [AB]	91	29F, 62M	84–141 months	learning disabilities	private elementary school for learning disabled children	hand, eye^1^, foot	Test Harris (^8^)	n.s.	**__**	n.s.	**__**	__
Dunlop et al., 1973 [12] [A]	30 (15N, 15D)	n.s.	103.3 months (N) 118.7 months (D)	reading disabilities	local district primary schools	hand, eye^2^	non-standardised (^8^)	Neale Reading Ability Test	**__**	**__**	__	__
Fagard et al., 2008 [15] [A]	42 (18GR1, 24GR5)	10F, 8M (GR1) 11F, 14M (GR5)	72 months (GR1) 120.4 months (GR5)	school population	regular public school	hand, eye^1^	non-standardised (^9^)	Alouette standardized reading test	**__**	**__**	**__**	**__**
Gates & Bond, 1936 [35] [A]	129 (64N, 65D)	n.s.	103.32 months	reading disabilities	n.s.	hand, eye^1^	non-standardised (^8^)	Gates Reading Diagnostic Test	**__**	**__**	Tests of word pronunciation	**__**
Harris, 1957 [36] [A]	561 (245D, 316N)	42F,274M (D), n.s (N)	120 months	reading disabilities	n.s.	hand, eye^1^	Test Harris (^8^)	n.s.	**__**	**__**	**__**	__
Hillerich, 1964 [37] [A]	400	n.s.	60 months 90 months at follow-up assessment	school population	kindergarten schools	hand, eye^1^	non-standardised (^8^)	California Achievement Test	**__**	**__**	**__**	California Short-Form Test of Mental Maturity
Kirk, 1934 [38] [B]	61	30F, 31M	high school	intellectual disability	state-funded institution for developmentally-disabled children	hand, eye^1^	non-standardised and Miles V-scope (^8^)	Gray Oral Reading Tests	__	__	__	__
Mahone et al., 2006 [39] [AB]	99	53F, 46M	36–70 months	school population	local preschools and day care centers	hand, eye^1^	non-standardised (^9^)	__	__	__	Peabody Picture Vocabulary Test	__
Muehl, 1963 [40] [B]	62 (23Y, 39O)	n.s.	48.7 months (Y) 58.2 months (O)	school population	cooperative preschools	hand, eye^1^	non-standardised and Miles V-scope (^8^)	non-standardised	**__**	**__**	**__**	**__**
Muehl & Fry, 1966 [41] [B]	40	21F, 19M	60–72 months	school population	cooperative preschools	hand, eye^1^	non-standardised and Miles V-scope (^8^)	Metropolitan, Achievement Test	**__**	Metropolitan, Achievement Test	**__**	__
Roszkowski et al., 1987 [42] [B]	58	29F, 29M	111 months	school population	catholic elementary school	hand, eye^1^, foot, ear^6^	DKSLD (^8^)	Educational Development Series	**__**	Educational Development Series	**__**	Otis-Lennon Test
Shaywitz et al. 1984 [18] [AB]	104 (32N, 37G, 35D)	104M	132 months	intelligence learning disabilities	public school	hand, eye^7^, foot^7^	non-standardised (^8^)	Woodcock-Johnson achievement battery	Woodcock-Johnson achievement battery	Woodcock-Johnson achievement battery	__	Wechsler Scale for Children-R
Smith, 1950 [43] [AB]	100	100M	120 months	reading disabilities	parochial and public schools	hand, eye^1^	non-standardised (^8^)	n.s.	**__**	**__**	**__**	**__**
Stephens et al., 1967 [44] [B]	89	45F,44M	first grades	school population	elementary schools	hand, eye^1^	non-standardised (^8^)	Metropolitan Reading Readiness Test	**__**	**__**	**__**	California Test of Mental Maturity
Thomson, 1975 [45] [A]	120 (60N, 60D)	n.s.	84.93 months (N) 84.82 months (D)	reading disabilities	primary schools	hand, eye^1^, foot, ear^6^	non-standardised (^8^)	Schonell Reading Test	**__**	**__**	**__**	__
Trussell, 1969 [46] [A]	75	36F,39M	first and second grades	school population	public elementary schools	hand, eye^1^	non-standardised (^8^)	Metropolitan Achievement Test	__	__	__	__
Ullman, 1977 [47] [B]	648	325F, 323M	66–149 months	school population	public schools	hand, eye^1^, foot^3^	non-standardised (^8^)	Wide Range Achievement Test-Revised	Wide Range Achievement Test-Revised	Wide Range Achievement Test-Revised	__	Lorge-Thorndike Intelligence Test
Witty & Kopel., 1936 [48] [A][B]	200 (100N,100D)	34F, 66M (N) 34F, 66M (D)	120.4 months (N) 108.2 (D)	reading disabilities	public schools	hand, eye^1^	non-standardised (^8^)	Metropolitan Achievement Test	**__**	**__**	**__**	**__**

Note: [A] Absolute crossed laterality. [B] Relative crossed laterality. [AB] Absolute or relative crossed laterality. (A) Advanced group. (N) Normal group. (D) Disabled group. (GR1) First grade. (GR5) Fifth grade. (Y) Young group. (O) Old group. (G) Gifted group. (n.s.) Not specified. (F) Female. (M) Male.

^(1)^ Sighting task.

^(2)^ Sighting task and binocular task combined.

^(3)^Bilateral task.

^(4)^Bilateral and unilateral task combined.

^(5)^ Dichotic listening task.

^(6)^ Unilateral listening task.

^(7)^ Not specified.

(^8^) Behavioural tasks.

(^9^) Behavioural tasks and questionnaire.

### Selection criteria

Studies were selected for inclusion in this review if they met the following criteria: (C1) They used one or more lateral preference tasks for at least two of the following parts of the body: hand, eye, foot, or ear; (C2) they included a measure of crossed laterality according to the *absolute* or *relative* operative definitions described below; (C3) they measured the impact of crossed laterality on academic achievement or intelligence; and (C4) they included participants between 3 and 17 years old.

### Definition of crossed laterality

Throughout this article, we adopted the term “crossed laterality” because it was the most frequent in the literature reviewed. However, it should be noted that some researchers employ different terms to refer to the same phenomenon, such as “confused laterality” [49], “mixed preference” [47], “mixed hand-eye” [40] or “mixed dominance” [18]. In this regard, it is worth mentioning that nowadays the term “mixed dominance” usually refers to the lack of preference with respect to the same type of limb or organ, not to the combination of preferences of two different types of limbs or organs (i.e., “mixed handedness” refers to an unclear preference of the right or left hand).

There is little consensus on the operative definition of crossed laterality through the reviewed literature. To address this problem, we established two operative definitions. *Absolute* crossed laterality refers to always using the same opposite sides of the body while performing different tasks with any combination of hand, eye, foot, or ear. In contrast, *relative* crossed laterality refers to the marked (but not exclusive) preference for using the same opposite sides of the body while performing different tasks. For example, a child whose dominant eye is the right in 80% of the observations and whose dominant hand is the left in 80% of them would not be considered crossed-lateral according to the first operative definition, but would be considered crossed-lateral according to the second one.

*Absolute* and *relative* crossed laterality differ from *mixed* laterality in that the latter refers to an undistinguishable or equivocally determined preference for using either side of the body while performing different tasks [37]. To continue the example above, a child whose dominant eye is the right in 50% of the observations and whose dominant hand is the left in 50% of the observations would be considered mixed lateral according to our definitions and consequently would not comply with the inclusion criteria of this review. Studies that only measured mixed laterality or that conflated mixed and crossed laterality in their analyses were not included in this review. For instance, studies that considered laterality as a continuous variable, from totally right to totally left, were not included [10,25,50–55]. Similarly, studies where mixed and crossed laterality were merged were also excluded [9,49,56–60]. In either case, intermediate scores could refer either to genuine crossed laterality or to mixed laterality. In addition, studies that failed to report any measure of crossed laterality [13,61–74] or that failed to explain how they categorized crossed lateral participants were excluded [75–78].

### Data extraction and coding

The 26 studies included were summarized in terms of characteristics of participants and setting (e.g., public school, catholic school), measures of crossed laterality, academic achievement, and intelligence (see Table 1).

## Results

### Participant characteristics

The number of participants included in each study ranged from 30 to 890 (mean = 201.3; *SD* = 204.8). The age of participants ranged from 53 to 132 months (mean = 104.4; *SD* = 24.4). Ten studies did not report the gender of participants and one study only provided the gender of one of the groups compared (see Table 1). Among the remaining 16 studies, 984 participants were female (38.2%) and 1590 were male (61.7%). The majority of participants (54%) were selected solely on the basis of being enrolled in a specific school, 23.5% were selected based on the presence or not of reading difficulties, 5.4% were selected based on the presence or not of learning difficulties or low intelligence, and 17% were selected based on being born in a specific hospital. In addition, 37% of participants came from public schools, 6.9% from private schools and 5% from religious schools. In the case of the remaining 49% of participants, the type of school was not specified.

### Methods used to measure laterality

The final sample of studies included in this review used a wide variety of questionnaires and behavioural tasks to measure laterality, independently of the definition of crossed laterality adopted (i.e., absolute or relative). It is worth mentioning that not all the tests employed to assess laterality were equally valid and reliable. They differed substantially in terms of the quantity of tasks included as well as in terms of the availability of normative data. The studies included in this review also focused on different combinations of hand, eye, foot or ear to assess crossed laterality (see Table 1). It is worth underlining that the measure of each part of the body comprises specific peculiarities and that the type of test employed may sharply constrain the results. The study performed by [79] offers a comprehensive analysis of the measurement of lateral preference and an extensive analysis of the prevalence and interrelationship of lateral preferences among the general population. Detailed information about each type of test that appeared in the present study is offered in the Supporting Information.

### Qualitative analysis

#### Crossed laterality and reading skill

As can be seen in Table 1, 23 studies measured the association between crossed laterality and reading performance. Only four of them [11,12,31,41] detected a significant positive association between crossed laterality and reading performance (i.e., children with crossed hand-eye laterality performing significantly worse than children without crossed laterality). Furthermore, two studies obtained significant results in the opposite direction: Shaywitz et al. [18] found that crossed lateral children showed fewer serious problems in reading than uncrossed lateral children, while Stephens et al. [44] found that children with crossed hand-eye laterality performed better in reading than children without crossed laterality.

#### Crossed laterality and spelling

Two studies measured the association between crossed laterality and spelling performance (see Table 1). None of them found a statistically significant association.

#### Crossed laterality and arithmetic skills

Four studies analyzed the effects of crossed laterality in arithmetic performance (see Table 1). Only Muehl and Fry [41] reported a significant contribution of crossed dominance to arithmetic achievement. Specifically, children with consistent right preference in hand and eye obtained better results than children with right hand and left eye preference.

#### Crossed laterality and language

Four studies measured the association between crossed laterality and language in terms of vocabulary or articulation (see Table 1). None of them found a significant relationship between crossed laterality and language performance.

#### Crossed laterality and intelligence

Eleven studies analyzed the relationship between laterality and intelligence (see Table 1). Only one of these studies found a relationship between crossed laterality and intelligence. Specifically, Bishop et al. [30] found a significant association between crossed laterality and intelligence in a small subgroup of children classified as predominantly uncrossed or predominantly crossed according to reference eye.

#### Other results

Together with the outcomes described above, one of the studies included in this review [39] explored the association between crossed laterality and visual attention. The results showed no relationship between these variables. In addition, Conolly [34] explored the association between crossed laterality and learning disabilities. The results showed that crossed laterality was more prevalent in learning disabled children than in normal children.

### Quantitative meta-analysis

The 26 studies selected for the present systematic review rely on a wide range of methods to assess crossed laterality, academic achievement, and intelligence. Consequently, any quantitative synthesis of these studies must be taken with caution. However, to avoid relying exclusively on a vote-counting approach we conducted a meta-analysis of the effect sizes reported in these studies. Unfortunately, about half of them failed to report sufficient information to compute a valid effect size estimate. The remaining studies provided enough information to compute an effect size in the Cohen’s *d* scale or reported contingency tables that could be used to compute an odds ratio, which we converted to a Cohen’s *d* effect size using the equations provided by [80]. All the effect sizes we computed focused on the comparison of crossed lateral and uncrossed lateral participants, which means that some of the comparisons covered in the previous qualitative review, which focused in specific sub-groups of crossed-lateral or preferentially crossed lateral children, were not included in the meta-analysis.

Although we could only extract a single effect size from most studies, some of them contained sufficient information to compute several effect sizes. For instance, Bishop et al. [30], measured crossed laterality using two different measures of eyeness (sighting eye and reference eye) and they also measured two outcome measures, reading and intelligence. Furthermore, they reported results in a way that allowed crossed laterality to be conceptualized according to both our absolute and relative definitions. Accordingly, we were able to extract six equally valid effect sizes from this study. Six other studies also contributed with several effect sizes to our meta-analysis. More information about the procedure followed to compute each effect size can be found at the Open Science Framework, https://osf.io/akg3h/.

Given that some studies contributed several effect sizes, while others were represented by a single effect size, these studies cannot be integrated using a simple univariate meta-analysis, because this would give extra weight to studies reporting several effect sizes. To overcome this problem, we fitted a three-level random effects models, clustering effect sizes at the study level. All the statistical analyses were conducted with the metafor R package [81].

**Fig 2:**
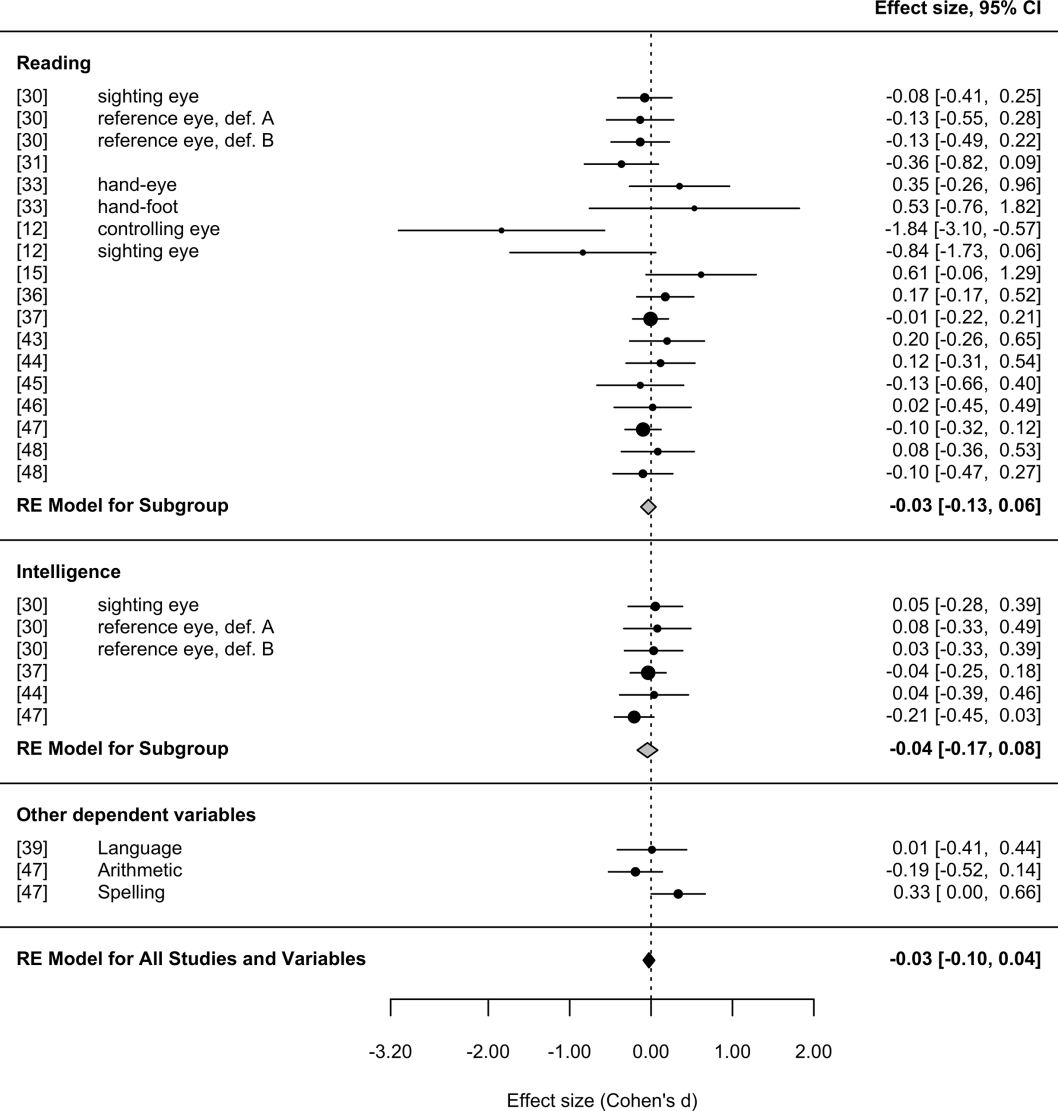
Forest plot of the effect sizes included in the meta-analysis.

All the effect sizes included in the general meta-analysis, with their corresponding 95% confidence interval, are depicted in Fig 2. Negative effect sizes denote a relative disadvantage of crossed lateral children over children without crossed laterality. As can be seen, only a handful of studies yield effect sizes that depart noticeably from zero, in either direction. The meta-analytic effect size for all studies, shown at the bottom, is *d* = -0.03, 95% CI [-0.10, 0.04], which is not significantly different from zero, *z* = -0.73, *p* = .46. Furthermore, the amount of heterogeneity across studies failed to reach statistical significance, *Q*(26) = 31.50, *p* = .21, suggesting that in principle the variation observed across studies can be attributed to chance alone. Fig 2 also presents the meta-analytic average for two subsets of studies that focused on the impact of crossed laterality on reading and intelligence. The meta-analytic estimates were *d* = -0.03 [-0.13, 0.06] and -0.04 [-0.17, 0.08], respectively, and in both cases failed to reach statistical significance, *z* = -0.67 and *z* = -0.69.

**Fig 3:**
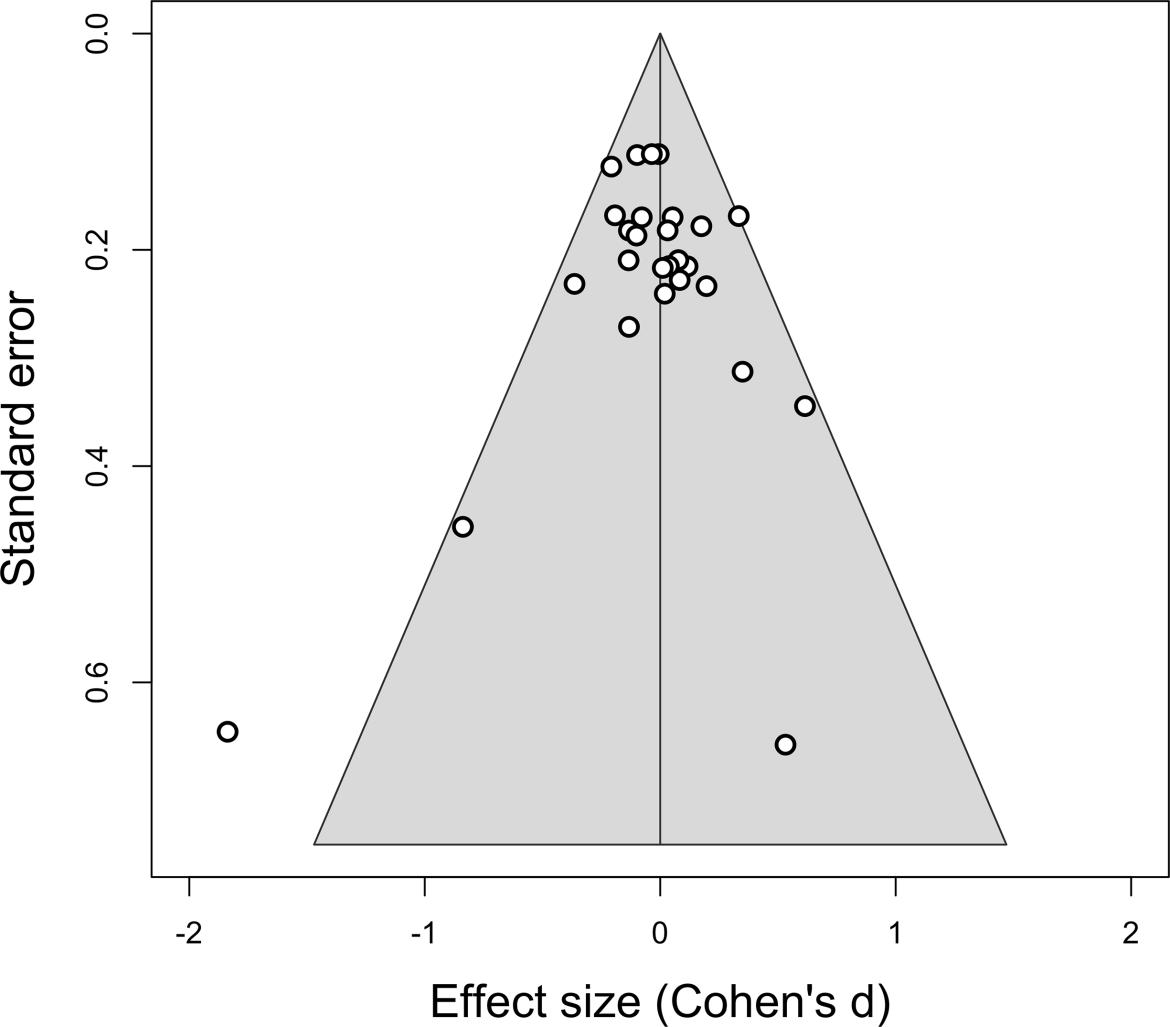
Funnel plot of the effect sizes included in the meta-analysis.

A funnel plot, with all the effect sizes plotted against their standard errors, is offered in Fig 3. All the effect sizes falling within the grey contour would be statistically non-significant in a two-tailed test. As can be seen, only studies with low precision (high standard errors) yield effect sizes that depart noticeably from zero, while studies comprising more participants tend to yield effect sizes consistently close to zero. Furthermore, the funnel plot shows that the distribution of effect sizes is clearly symmetrical, suggesting that the results of the meta-analysis are unlikely to be affected by publication bias.

## Discussion

The majority of the studies included in this review failed to find any relationship between the preference for different sides of the body when performing tasks that involved any combination of hand, eye, foot, or ear and performance in reading, spelling, arithmetic, language or intelligence tests. As explained in the qualitative analysis, some studies showed that crossed laterality was associated with lower scores in reading [11,12,31,41], arithmetic [41,42,47], intelligence [30] or other variables [34]. However, these results were not consistently replicated in other studies and sometimes they even failed to be replicated within the same study with different selection criteria or different dependent variables. Furthermore, the quantitative meta-analysis shows that among those studies reporting sufficient information to compute an effect size the average effect size was not significantly different from zero.

For instance, in the study of Dunlop et al. [12] the lower performance of children with crossed laterality was only observed when measuring eye dominance with the criterion of the controlling eye but not with the criterion of the sighting eye, which is the most widely used in the literature (see the S1 File and [82]). In addition, they measured the preferred hand, using a unique writing task, which has questionable validity for being frequently culturally biased [83]. It is also worth noting that the sample size of the study conducted by Dunlop et al. [12] is only 30 participants (the smallest sample in Table 1) and that the two groups of children (dyslexics and controls) came from very different educational and social backgrounds, without any attempt to match them in terms of age or other variables. In a study specifically designed as a replication of Dunlop et al., [12] Bishop et al. [30] failed to replicate these results with a much larger sample (*N* = 147), although as discussed below she did detect lower intelligence in a small subset of crossed lateral children.

Similar problems apply to the other three studies reporting a positive relationship between crossed laterality and reading problems. In the study by Bryden [11] the relationship between crossed laterality and lower reading performance was confined to boys with an intermediate or low reading achievement in relation to their IQ. In the case of girls, no relationship between crossed laterality and reading disabilities was observed under any assumption. In a study with only 40 participants (the second smallest study in Table 1, after [12]), Muehl and Fry [41] found that uncrossed lateral children (right-hand and right-eye) performed better in a reading task than crossed lateral children (right-hand and left-eye) and left-eyed children. However, due to the reduced number of children with left-hand and right-eye laterality and left-hand and left-eye laterality, the authors could not determine whether crossed laterality or eyeness was the outcome associated to lower reading achievement. The same problem applies to the study conducted by Brod and Hamilton [31], who actually concluded that the poor performance of crossed lateral children was not due to crossed laterality itself but to the fact that most children in this sample were left-eye dominant.

Furthermore, two studies reported better reading skills in crossed-lateral children than in control participants. Specifically, Shaywitz et al. [18] found that children with crossed laterality showed fewer problems of a severe nature in reading. Similarly, Stephens et al. [44] found that children with crossed laterality had a tendency to perform better in reading than those with an uncrossed laterality pattern, although this trend failed to reach full statistical significance. As in other studies finding a significant relationship between crossed laterality and reading performance, these two studies relied on relatively small samples of only 104 and 89 participants, respectively.

Overall, the collective weight of this evidence does not support the conclusion that there is a reliable association between crossed laterality and reading performance. Significant (either positive or negative) associations between both variables were observed predominantly among the smallest studies and only in some of the dependent variables or in small subgroups of participants. The majority of studies, including those with the largest sample sizes, found no relationship between crossed laterality and reading skills. This conclusion is further supported by the results of the meta-analytic synthesis, as can be seen in Fig 3.

A small number of studies reported positive correlations between crossed laterality and other variables related to academic achievement or intelligence. Muehl and Fry [41] detected that uncrossed lateral children (right hand and right eye) obtained better results in arithmetic tasks than crossed lateral children (right hand and left eye). However, as explained above, the authors could not establish whether crossed laterality or eyeness was the outcome related to poorer performance. Additionally, the small sample size of this study raises suspicions about the reliability of this finding, bearing in mind that the other three studies exploring this association failed to detect a significant result.

One study detected a significant association between crossed laterality and intelligence [30]. Interestingly, in this study there were no significant differences in intelligence between uncrossed- and crossed-lateral children. Rather, the significant differences found by Bishop et al. [30] were entirely due to the poor performance of a small group of children (*N* = 31) with ‘predominantly uncrossed reference’ or ‘predominantly crossed reference’, that is to say, children meeting our relative definition of crossed laterality. Far from concluding that there is a reliable association between crossed laterality and intelligence, the author herself concluded that ‘this study finds no evidence that “crossed reference” [i.e., laterality] has any particular significance.’ (p. 665). Furthermore, this trend fails to reach statistical significance when all participants are considered, as confirmed by the confidence intervals reported in Fig 2.

Likewise, one study found that the proportion of children with crossed laterality was higher among learning disabled than among normal subjects [34]. However, the author did not specify the tools employed to classify children as learning disabled. In addition, she did not use a control group, but simply compared her results with the pattern observed in two earlier studies with completely different and unmatched samples [84].

Finally, no study detected an association between crossed laterality and spelling or language. Overall, the fact that the majority of studies failed to observe significant differences and that the few studies with significant findings yielded inconsistent results suggests that the significant effects obtained in children with crossed laterality are likely to be unreliable. This conclusion is consistent with the results of similar studies that were excluded from this review because they failed to provide an operative definition of crossed laterality or because they treated crossed and mixed laterality indistinctly (e.g., [16,56,57,59,60,85], but see [13]). Similarly, these results are also consistent with previous non-systematic reviews done in the field of laterality [86,87].

This review also highlights the substantial degree of heterogeneity that characterises studies of crossed laterality. In particular, there is little or no consensus on the operative definition of crossed laterality. While some studies establish strict criteria to define this phenomenon, other studies are quite flexible and vague or do not provide any operative definition of the concept at all (e.g., [60]). Furthermore, the tools employed to measure laterality are quite miscellaneous (see the Supporting Information) and, in many cases, the authors do not report indices of reliability and validity for their measures [12,15,39,45]. Finally, there is great diversity among the characteristics of the samples and the outcomes measured. Taken together, these limitations hinder any progress in this area of research.

The results of this study have important implications for education. The lack of strong evidence in favour of a relationship between crossed laterality and academic achievement calls into question the fact that educational psychologists spend time measuring crossed laterality and, even more, that crossed laterality should be the object of direct intervention to ameliorate learning disabilities. These results, in turn, echo the voices that have been raised previously against evaluations and interventions of this kind [49,86,88]. At present, there is no solid evidence that justifies the adoption of interventions addressed at treating laterality by education practitioners, let alone the use of them as a replacement for evidence-based interventions directly aimed at the target difficulties.

## Supporting information

S1 Table PRISMA checklist. (DOC)

S1 File (DOCX)
