# Supplementary material for: Is crossed laterality associated with academic achievement and intelligence? A systematic review and meta-analysis
Source: PLoS One. 2017 Aug 28;12(8):e0183618. doi: 10.1371/journal.pone.0183618 (PMC5573212; doi:10.1371/journal.pone.0183618)
Supplement: S1 File — (DOCX) [file pone.0183618.s002.docx]

**SUPPORTING INFORMATION**

Is crossed laterality associated with academic achievement and intelligence?

A systematic review and meta-analysis

Marta Ferrero, Gillian West, & Miguel A. Vadillo

As explained in the main text, the studies included in the present systematic review relied on a wide variety of tests to measure the lateral dominance of different parts of the body, not all of them equally reliable and valid. There is abundant literature about the differences between questionnaires and behavioural tasks, mainly in relation to the measure of handedness. Although the lateral preferences reported on questionnaires tend to agree with behavioural measures –and test-retest reliability is satisfactory in both cases– the validity of the former seems to be more limited [1] A detailed description of all the behavioural tasks used in each study is beyond the scope of the present article. However, in the following sections we offer a brief description of the tests and measures employed in the studies included in the systematic review.

*Hand lateral preference*

Several studies give different reasons in favour of behavioural measures of handedness over questionnaires [2-4]. Among behavioural tasks, some tests measure hand preference, while others focus on the relative proficiency of each hand. The former give equal weight to preference responses for a collection of different activities (e.g., Harris Tests of Lateral Dominance), while the latter measure the degree of handedness in terms of an internally consistent continuum (e.g., the reaching test). The relationship between hand preference tests and right-left performance is not clear [5] and, depending on the tasks used to assess both parameters, what is considered as handedness by different studies may represent different traits [6].

*Eye lateral preference*

We can distinguish three criteria to define eye dominance: the eye with the better visual acuity, the eye that predominates during binocular rivalry, and the eye used for sighting [7]. Acuity tests consist on determining the smallest letters a person can read on a standardized chart [8]. Binocular rivalry tests consist on presenting a person two rivalling stimuli through a stereoscope to determine the eye for which a stimulus is predominantly reported [9]. Sighting tests consist on forcing a person to look straight ahead with one eye through a telescope, or through a hole in a piece of cardboard to determine which is his/her preferred eye [10].

Apparently, these three factors are independent and, consequently, eye dominance measured with one criterion does not correlate well with that measured with the other criteria [9] Among these measures, the sighting-dominant eye comes closest to satisfying the assertion that there is a dominant eye for a given test [9] and, not surprisingly, it is the most frequently used criterion to determine eye dominance. Several sighting tests include tasks that require the use of one hand.

Similarly, some questionnaires ask about handedness and immediately afterwards go on to ask about eyeness. Both procedures have been shown to increase a tendency for the dominant hand to influence the measurement of the dominant eye. Some experimenters have attempted to remove all potential biases when measuring eye preference [2]. Unfortunately, the majority of studies included in this review do not give information about this particular concern so we cannot made any distinction based on this criterion.

*Foot lateral preference*

There are two ways to assess foot preference: via bilateral tasks, in which the two limbs are required (e.g., kicking a ball), and via unilateral tasks, in which only one limb is required (e.g., standing on one foot). The foot chosen for a bilateral task is independent of the foot chosen for a unilateral task. In other words, the foot preference may depend on the nature of the task used [11]. Due to the high reliability observed in the foot chosen for a single action, but not in the foot chosen for keeping balance, the majority of test batteries define the dominant foot as the one chosen in a bilateral task to act on an object while the other foot preserves stability.

*Ear lateral preference*

There are two kinds of task to measure ear lateral preference: dichotic listening tasks and unilateral listening tasks. Dichotic listening tasks involve the simultaneous presentation of two different lists of audio stimuli, one to each ear. Unilateral tasks involve the selection of right or left ear to listen to a stimulus. While dichotic listening tests always imply the use of behavioural tasks, unilateral tests can rely on either behavioural tasks or questionnaires. As in other measures of laterality, ear preference may vary according to the nature of the task used [12]. Although some studies have obtained similar results in both types of tasks [13], studies that have employed dichotic tasks have mainly reported a right-ear preference [14,15], while studies that have employed unilateral tasks have frequently reported a left-ear preference [12,16].

**References**

[1] Raczkowski, D., Kalat J. W.,Nebes, R. Reliability and validity of some handedness questionnaire items. Neuropsychologia.1974;12: 43-47.

[2] Bourassa DC, McManus IC, Bryden MP. Handedness and eye-dominance: A meta-analysis of their relationship. Laterality. 1996;1: 5-34.

[3] Carlier M, Doyen AL, Lamard C. Midline crossing: Developmental trend from 3 to 10 years of age in a preferential card-reaching task. Brain Cognition. 2006;61: 255-261.

[4] Dellatolas G, Curt F, Dargent-Paré C, De Agostini M. Eye dominance in children: A longitudinal study*.* Behav Genet. 1998;28: 187-195.

[5] Fagard J, Corroyer D. Using a continuous index of laterality to determine how laterality is related to interhemispheric transfer and bimanual coordination in children. Dev Psychobiol. 2003;43: 44-56.

[6] Ocklenburg S, Beste C, Arning L. Handedness genetics: Considering the phenotype. Front Psychol. 2014; 5: 1300.

[7] Howard IP. Seeing in depth: Vol. 1. Basic mechanisms. Toronto: I. Porteous; 2002.

[8] Pointer, JS. Sighting dominance, handedness, and visual acuity preference: three mutually exclusive modalities? Ophthal Physl Opt. 2001*;21*: 117-126.

[9] Mapp AP, Ono H, Barbeito R. What does the dominant eye dominate? A brief and somewhat contentious review. Percept Psychophy. 2003;65: 310-317.

[10] Fagard J, Monzalvo-López K, Mamassian P. Relationship between eye preference and binocular rivalry, and between eye-hand preference and reading ability in children. Dev Psychobiol. 2008;50: 789-798.

[11] Hart S, Gaddard C. Brief communication: Bilateral footness and task complexity. Int J Neurosci. 1996;88: 141-146.

[12] Seeman J, Surwillo WW. Ear preference in telephone listening. Percept Motor Skill. 1987;65: 803-809.

[13] Porac C, Coren S. *Lateral preferences and human behaviour*. New York: Springer-Verlag; 1981.

[14] Carr BM. Ear effect variables and order of report in dichotic listening. Cortex. 1969;5: 63-68.

[15] Kimura D. Functional asymmetry of the brain in dichotic listening. Cortex. 1967;3: 163-178.

[16] Survillo WW. Ear asymmetry in telephone-listening behaviour. Cortex. 1981;17: 625-632.
